# A global framework for integrating public health into wellbeing: why a public wellbeing system is needed

**DOI:** 10.3389/fpubh.2025.1454470

**Published:** 2025-02-06

**Authors:** László L. Lippai, Klára Tarkó, Attila Tanyi, Zsófia Kollányi, Mária Arapovics, József Vitrai

**Affiliations:** ^1^Institute of Applied Health Sciences and Environmental Education, University of Szeged, Szeged, Hungary; ^2^MTA-SZTE Health Promotion Research Group, Szeged, Hungary; ^3^Department of Philosophy (IFF), UiT The Arctic University of Norway, Tromsø, Norway; ^4^Corvinus Institute of Advanced Study (CIAS), Corvinus University, Budapest, Hungary; ^5^Department of Economics, Faculty of Social Sciences, ELTE Eötvös Loránd University, Budapest, Hungary; ^6^Department of Social Work, Faculty of Social Sciences, ELTE Eötvös Loránd University, Budapest, Hungary; ^7^Department of Preventive Health Sciences, Faculty of Health and Sport Sciences, Széchenyi István University, Gyor, Hungary

**Keywords:** quality of life, psychological wellbeing, public health, happiness, social determinants of health

## Abstract

There is a growing focus on public health initiatives that prioritize wellbeing. The main question of our study is whether this, in its current form, can really represent a new response to the challenges of previous strategies, or whether there is a greater chance that it will essentially reproduce the problems associated with the paradoxical situation of public health. Based on a review, analysis and evaluation of the literature on wellbeing in public health, we outlined the foundations of a new meta-theory of wellbeing and a possibility for its social application. In our view, wellbeing is seen as a social representation of a combination of positive and negative freedom of choice concerning the quality of everyday life, used in a positioning process involving both individual and collective aspects. Health is a particular aspect of the social representation and positioning of wellbeing, which encompasses aspects of the physical, psychological, social and spiritual functioning of individuals. The wellbeing meta-theory also opens up the possibility for more effective solutions to the social challenges related to wellbeing and salutogenetic health. It underscores the importance of the need for a dedicated social subsystem where the goals and organizational culture of the organizations involved are focused on wellbeing and health promotion. In our study, we consider this to be the Public Wellbeing System (PWS). Our conclusion is that the development and operation of a new set of institutions—the Public Wellbeing System (PWS)—based on the co-production of services that meet the needs and demands of society, and dedicated to the promotion of wellbeing, may provide an opportunity to overcome the public health paradox.

## 1 Introduction

It is becoming increasingly clear that our approach to public health needs to be renewed. This is evidenced, for example, by the unstoppable obesity epidemic (1), persistent health inequalities (2), or the critique of the coronavirus epidemic (3). The growing recognition of the links between health and sustainability (4), biodiversity (5), ecosystems (6), and the climate crisis (7) makes it more urgent than ever to develop new approaches that address these issues.

This is not the first time in the history of public health that such a need for renewal has been at issue. The need for renewal is driven by accumulated experience and new knowledge, and some experts believe it can be traced to distinct phases in the history of public health. A group of researchers on the European side of the Atlantic label these phases' waves' (8) and claim that we are already in their fifth, while on the other side of the Atlantic, they are labeled as major public health improvements and we are said to be in the third (9). There is no consensus on the use of labels. This is evidenced by the fact that the Culture for Health label, referred to as the fifth wave in the UK, no longer appears on NHS websites and in NHS documents (10). And how successful has the implementation of the proposed changes, labeled Public Health 3.0, been in the US? In 2021, DeSalvo, the “mother” of Public Health N3.0, and colleagues proposed almost identical recommendations to those made 7 years ago for policymakers to consider as the nation charts a course for the post-pandemic era (11).

It can also be observed that the development of public health is closely linked to the integration of results from other disciplines. In the field of psychology, the integrative theory of behavior change, the spread of COM-B (12), and the health insights of positive psychology (13) should be highlighted. Systems science has had a truly revolutionary impact on public health by revealing the characteristics of complex systems and developing a methodology to study them (14). It has also stimulated the health sciences, which have developed a health model based on a systems approach (15). In sociology, the methodological experience of action research (16) and the concept and practice of “citizen science” (17), in cultural anthropology and cultural studies the recognition of the role of culture in health behavior (18, 19), in public policy the identification of social problems as “wicked problems” (20) that seem intractable, have significantly changed the way public health problems are understood and addressed. Knowledge of implementation science has led to further fundamental changes in the implementation and evaluation of interventions to address public health problems (21).

Despite these achievements, the need for public health renewal suggests that public health efforts to date have only been partially successful. One reason for this is the paradoxical social situation of public health, i.e., the fact that the social challenges for which public health is responsible can only be partially addressed by a public health system based on the perspective, priorities, and scope of the healthcare system (22, 23). To resolve this paradox, *health promotion* has been proposed [again, see (24)], to ensure social influence among the “healthy” to adopt healthier lifestyles and to have the necessary and supportive living conditions (22). What was originally a Canadian initiative was taken to an international level by the WHO's Ottawa Charter in 1986. The Charter established the concept of health promotion as a new approach to public health. The concept was developed based on the evidence-based recognition that health, and therefore its promotion, is not only a healthcare function but also an individual and community endeavor that goes beyond a healthy lifestyle to include wellbeing (23).

More than three decades on, however, it is clear that the social organization of health promotion that was the hallmark of the Ottawa Charter has only been partially achieved. The problems are well illustrated by the WHO (25) assessment of school health promotion, which shows that although the concept has been partially implemented in some countries, health promotion is still not an integral, sustainable part of the functioning of the public education system. The scope and lack of pedagogical competencies of public health professionals focusing on public education do not allow them to sufficiently integrate health promotion into the daily life and professional tasks of public education organizations, beyond biomedical public health tasks. Mostly, education professionals do not feel that they have this responsibility, and do not understand what right and basis they have to promote health (25). Similar issues arise in all settings relevant to health promotion: the workplace, cultural and community settings, local communities, families, and policy-making.

More recently, WHO has sought to address the public health paradox through its “Health in All Policies” initiative. In our view, and in line with the analysis of Greer et al. (26), this program has also reached its limits. The main reason is that “engaging other sectors has often proven difficult. In some cases, policymakers have supported measures that are damaging to health, often drawing on overly narrow economic arguments that prioritize short-term benefits to some sectors over long-term costs to society—for example, by promoting polluting extractive industries. Some policymakers have reservations that Health in All Policies means health ministers expect other people to solve their problems” (27).

We are currently witnessing the emergence of a new WHO strategy to address the public health paradox with a focus on wellbeing, marked by new foundational documents such as the Geneva Charter for Wellbeing (28) or the Global Framework providing guidelines for the promotion of wellbeing (29). It is worth taking a closer look at whether this wellbeing-focused public health initiative is indeed a new response to the challenges of previous strategies, or whether it is more likely to be a new incarnation of the problems associated with the public health paradox.

In this context, our study has three objectives. We examine the theoretical and practical implications of the wellbeing narrative on which the current WHO wellbeing initiatives are based. In other words, is it sufficient to replace the word 'health' with 'wellbeing' to bring about the public health revolution that the WHO hopes for?

Based on this analysis, we propose a wellbeing meta-theory as an alternative approach to the public health paradox. Finally, we present the possibilities and limitations of the social operationalization of this meta-theory.

## 2 Steps toward a wellbeing meta-theory

### 2.1 The public health narrative of wellbeing—current situation

Although there have been many attempts to conceptualize wellbeing in different disciplines such as psychology, economics, sociology, and philosophy, there is still no consensus on the theoretical basis of the concept (30–32). The problem of definition has been present and pronounced in the wellbeing literature for decades, i.e., there is currently no consensus about the definition of wellbeing. Typically, the authors of studies on wellbeing establish this fact and then go on to present a new concept of wellbeing that is worthy of consensus (30, 33–35).

In the *health sciences*, wellbeing has been considered for some time as a possible broader way of thinking about and exploring human health. It is essentially used to counterbalance a narrower disciplinary approach focused on individual physical functioning (36).

The WHO Glossary of Health Promotion defines wellbeing as “Wellbeing is a positive state experienced by individuals and societies. Similar to health, it is a resource for daily life and is determined by social, economic, and environmental conditions. Wellbeing encompasses quality of life, as well as the ability of people and societies to contribute to the world with a sense of meaning and purpose. Focusing on wellbeing supports the tracking of the equitable distribution of resources, overall thriving, and sustainability. A society's wellbeing can be observed by the extent to which they are resilient, build capacity for action, and are prepared to transcend challenges” [(37), p. 10].

This definition provides an opportunity to channel several findings from the multidisciplinary wellbeing research of recent decades into public health. It emphasizes the salutogenetic aspect of wellbeing (38), which it sees as a subjective state of the individual or society. It stresses the importance of resource orientation and social determinants. In addition to the quality of life as an objective variable, subjective variables such as meaningfulness (38), purpose, or human capacity (39) are also emphasized. By emphasizing equity in the distribution of resources, growth, and sustainability, WHO also links social values to the concept of wellbeing (4, 39). In this approach, social wellbeing is made visible through resilience, agency, and willingness to change.

In summary, the glossary (37) essentially interprets wellbeing as the positive side of the health-disease continuum, emphasizing its subjective and social aspects without specifying how the two concepts differ. But can such a narrative provide an interface between the scholars in psychology, economics, sociology, philosophy, and many other disciplines, and the society outside the health-care system?

In our view, it cannot. By defining wellbeing as a concept “like health,” WHO inadvertently and unintentionally privileges health sciences and healthcare (again) at the expense of other disciplines and specialties. In essence, it expects other sectors to take appropriate action to achieve wellbeing, as WHO has done with the slogan 'health in all policies'. With such an approach, wellbeing cannot become a field for multidisciplinary and multisectoral cooperation. It cannot achieve this even though, thanks to the diversity resulting from its integrative aspirations, public health could be an excellent *mediator* of cooperation.

However, in the absence of a partnership approach, there is a risk that this mediating role, even under the guise of “wellbeing,” will not be accepted by society and that the public health paradox will be effectively reproduced, i.e., that society outside the health sector still does not feel responsible or competent to do anything about health.

This is not just a “theoretical” question. The basic WHO documents on wellbeing (28, 29) mentioned above also use the glossary definition (36), so it may be crucial from a practical point of view to recognize the limitations of this definition and to analyze possible alternatives.

In our study, we've put forward two proposals to overcome the public health paradox and move beyond the limitations of the current WHO definition of wellbeing. On the one hand, we propose a meta-theory of wellbeing that could provide an opportunity to synthesize wellbeing research that has been treated separately by disciplines. On the other hand, we also propose a public wellbeing system based on the wellbeing meta-theory, which could provide an opportunity to coordinate professional efforts to improve wellbeing, which have so far been separated across social sectors.

### 2.2 The assumptions of scientific wellbeing narratives

A systematic review of the diverse and eclectic literature on wellbeing in philosophy, psychology, economics, health, sociology, and many other disciplines is beyond the scope of this paper. However, after reviewing some key disciplinary summaries and conceptualizations of the topic (30–36, 39–41), we have found that the very different narratives of wellbeing in different disciplines share elements that can be considered common across disciplines. The starting point for developing our meta-theory of wellbeing was therefore to identify a set of often unspoken premises that may be common to most of the existing models of wellbeing. We also explored whether they could be built upon in the development of a meta-theory of wellbeing. For our study, two such premises proved relevant to our analysis.

*Premise 1: The principle of “the more is better” prevails when it comes to wellbeing*. In our view, the majority of scientific narratives (30–36, 39–41) focus mainly on the idea that an increase in wellbeing requires a quantitative increase in some material, psychological or social dimension. There is a need for more material goods, health, happiness, cultural capital, social cohesion, etc. to increase wellbeing.

This assumption, which seems logical at first glance, obviously has its limitations, which we believe are not sufficiently taken into account. In our view, relatively little attention is paid to the so-called Easterlin paradox, which explores the principle of diminishing marginal utility in a broader context, as it is known from economics. The essence of this phenomenon, described in the mid-1970s, is that an increase in income is associated with an increase in happiness only up to a certain point. After a turning point, the increase in income is no longer associated with an increase in happiness, the two phenomena become independent of each other. This turning point may be different not only for each individual but also for each culture and nation (42).

It is also worth considering that in many individual or social situations, the application of “the more is better” principle *can cause significant individual and collective harm*. This is illustrated, for example, by the psychological model of the *hedonic treadmill*. This means that the effect on happiness of any change in our circumstances is only temporary because the psychological adaptation to change happens very quickly. Even in the case of large lottery winnings, and relatively large changes in health or even living conditions, there is empirical evidence that their effects are temporary and that happiness levels return to their pre-change baseline after a while (43).

This creates many public health traps at both individual and collective levels. On the individual level, the importance of the hedonic merry-go-round in addiction is striking. Their danger is illustrated by the *tolerance* that develops in the misuse of psychoactive substances, which indicates a correlation between increasing doses of substances and decreasing psychoactive effects [e.g., alcoholism, drug abuse, etc.; cf. (44)]. On the collective level, it may also be an individual component of the overconsumption that drives economic growth to levels that threaten sustainability, alongside the socio-economic factors that fuel it [see (4, 36)].

It probably also matters *when* the more is better. In Ainslie's model of behavioral economics (45), the early satisfaction of intrinsic desires before their optimal time is motivated by hyperbolic discounting, i.e., intertemporal decision processes. It is the surprise, the novelty, that can confirm the desire, which is often accompanied by unbridled emotions. It is only through the will that one can overcome the unbridled emotions of inner origin, which, if too successful, can reduce the power of the reward associated with that emotion (45).

And even when it comes to *freedom of choice*, it is not clear that more is better. An example of this is Elster's behavioral economics analysis of “weakness of will” (46), which analyses decision situations from a behavioral economics perspective where *less is better*. There are also examples of someone—like Odysseus, who listened to the sirens and was tied up well in advance—taking preliminary strategic steps “against himself” to make it difficult or even impossible to fail the implementation of the rational alternative. Therefore, according to Elster, there are situations where it is desirable to limit the consumer's choice (46).

*Premise 2: Individuals' subjective assessments of wellbeing are “perfectly reliable” from both an individual and a collective perspective*. Another common point of scientific narratives of wellbeing (30–36, 39–41), especially in the case of individual- or collectively-oriented models that emphasize subjective wellbeing, maybe that the most reliable measure of wellbeing is individual assessment.

However, several findings from cognitive psychology, decision theory and social psychology warn against this. Human perception is shaped by perceptual and attentional constraints, schema categorization biases, decision heuristics, and attribution biases, as well as influences of emotion, cognitive dissonance, reference group norms, and peer comparison, to name a few variables from a long list (47). And people's judgments, decisions, and evaluations of situations are shaped by their perceptions of objective reality, not by objective reality itself. Moreover, even with an accurate perception of objective reality, we cannot be sure that individual wellbeing simply adds up to a collective construct of wellbeing.

This does not mean that there is no need to explore subjective wellbeing. On the contrary, it is very important to explore the perceptions and distortions of individual wellbeing to help individuals and communities identify and address the perceptual and evaluative biases that affect their wellbeing.

The search for wellbeing is therefore a reflective process and has implications for wellbeing itself. In the scientific modeling and research of wellbeing, individuals, families, small groups, organizations, local communities, professional communities, and societies not only communicate about wellbeing but also shape it through their reflections.

In the scientific understanding of wellbeing, it is also worth remembering that the act of research itself changes the object of research (48). It is, therefore, preferable to define wellbeing research as a process of promoting a systemic, multi-level (individual, family, small group, organizational, local community, and societal) self-reflection on wellbeing.

The WHO concept of wellbeing (37) is also permeated by these two premises and their associated dilemmas. ‘The more is better' principle is also reflected in the promotion of quality of life resources, resilience, and empowerment. There is also a reference to the importance of sustainability, but no guidance on how to reconcile growth and sustainability.

Similarly, premise 2 is also reflected in the WHO definition. The glossary distinguishes between individual and societal levels of wellbeing and identifies their main dimensions of assessment, but does not specify the reference points for addressing their consensual nature and dynamics.

## 3 A possible meta-theory of wellbeing

We argue that the above two assumptions and their difficulties in scientific narratives of wellbeing, including the WHO definition of wellbeing, become more manageable *when wellbeing is seen as a social representation of a combination of positive and negative freedom of choice regarding the quality of everyday life*. This concise definition of wellbeing can be elaborated as follows.

### 3.1 Wellbeing as social representation

*Social representation*, as defined by Moscovici (49), is the multidimensional space resulting from the creative interaction between social-societal and individual cognition, stretched by the concepts of different phenomena and shaping individual and collective action. Social representation therefore includes social factors that derive from social status, role and culture as well as individual perception. However, it focuses primarily on the actual outcome of the dynamic interaction of these factors. It moves from the social relations established in interpersonal relationships to individual reactions and attitudes. It represents the creative process in personal and mass communication as well as the cognitive structure that is present in it (49). Doise sees social representation as a social-societal metasystem of individual cognition that shapes individual cognition. It provides reference dimensions for individual cognition, but unlike social norms, it does not prescribe an individual's point of view. This metasystem provides a common perspective along which individual and group differences can be articulated (50). The social representation of wellbeing is thus a multidimensional space, stretched by the concepts of wellbeing at the individual, family, local community or the whole society level. The notion of social representation provides the flexibility needed to scientifically capture the gender, cultural, ethnic and social diversity and over time changes in the concept of wellbeing.

### 3.2 The process of wellbeing positioning

In the meta-theory of wellbeing, we propose, that wellbeing is a dynamically changing social representation used in a positioning process that includes both individual and collective aspects.

*Wellbeing positioning* is the process by which individuals or communities evaluate their current situation using a dynamically changing frame of reference of the social representation of wellbeing. In essence, then, the individual or community situates itself in the multidimensional space in which, through the social representation of wellbeing, their complex set of aspects related to the quality of everyday life is *currently* constructed.

At the individual level, this complex process, which includes cognitive, affective, and behavioral aspects, can also be referred to as *subjective wellbeing*, the phenomenological experience of which is perhaps best captured by Antonovsky's concept of coherence (38), and the mood aspect of which could be expressed by the concept of happiness (30).

However, wellbeing positioning also takes place concerning the family, the organization, the local community, the region, society, and humanity, in a way that affects all or some dimension of its multidimensional space. Subjective wellbeing is therefore an important form of wellbeing positioning, but the positioning process at other levels can be just as important.

### 3.3 Freedom of choice is the crystallization point of wellbeing

In our meta-theory, which builds on the work of Amartya Sen, *freedom of choice* is at the center of representation and valuation processes in the social representation of wellbeing (39). In our concept, the social representation of wellbeing refers not only to the criteria that individuals, families, communities, and societies *currently* use to account for the factors that limit or enhance their *freedom of choice*. It also includes how individual and collective considerations are constantly shaping the social and societal metasystem itself. On the collective side, the social representation of wellbeing can be influenced by scientific models and research findings as well as by current political, religious, and cultural discourses. However, in the dynamics of the construction and use of social representation, individual perceptions, experiences, and emotional relations have as much role and significance as the social-societal communication of individuals.

Inspired by Berlin's (51) work on political freedom in conceptualizing freedom of choice, we believe that freedom of choice, which is also the basis for understanding and assessing wellbeing, should be further analyzed in terms of *positive and negative freedom factors*. These two independent (orthogonal) factors provide a good approximation of the multidimensional space of the social representation of wellbeing. In other words, these factors bring the many different aspects used to assess wellbeing into a common denominator and can be used to illustrate and interpret wellbeing positioning to a good approximation.

In our metatheory, the *positive freedom of choice* factor of the social representation of wellbeing is constituted by all aspects of everyday quality of life whose availability, possession or affordability is represented as important and beneficial for individuals, communities or society. The factor of positive freedom of choice is therefore constituted by the positive (achievable) preference structure in the social representation of wellbeing, and its extent is the extent to which individuals, communities or society perceive that these positive preferences are successfully implemented in everyday life. This positive preference structure, which includes both existing and desired aspects and reflects both individual and collective perspectives, is a constantly changing construct due to individual, community and societal changes.

A key observation is that, from a research perspective, the positive freedom of choice factor can be segmented according to the objective and subjective consequences associated with a given value of positive freedom of choice (see chapter The positive freedom of choice factor of wellbeing).

In our metatheory, the *negative freedom of choice* factor of the social representation of wellbeing includes aspects of everyday quality of life whose *absence or avoidance* is represented as important and beneficial for individuals, communities or societies. The negative freedom of choice factor is thus constructed from negative (avoidable) preferences for the quality of everyday life, which is also dynamically shaped by individual, community and societal changes.

The factor of negative freedom of choice in the social representation of wellbeing is thus the negative preference structure of individuals, communities and societies, and the extent to which they are perceived to be able to enforce these negative preferences in their everyday lives. Individual aspects (e.g., self-control) can play a role in whether negative preferences are enforced, as can community (e.g., cultural norms, group norms) or social aspects (e.g., the legal system) that help or hinder them.

Again, it is important to note that from a research point of view, the negative freedom of choice factor is also considered segmentable (see chapter The negative freedom of choice factor of wellbeing).

These factors are analyzed in more detail below. The dynamically changing measures of wellbeing, referred to as positive and negative freedom of choice, will also be the basis for individuals, communities and societies to assess their own position, as presented in our chapter on the wellbeing positioning process.

### 3.4 The positive freedom of choice factor of wellbeing

**Figure 1:**
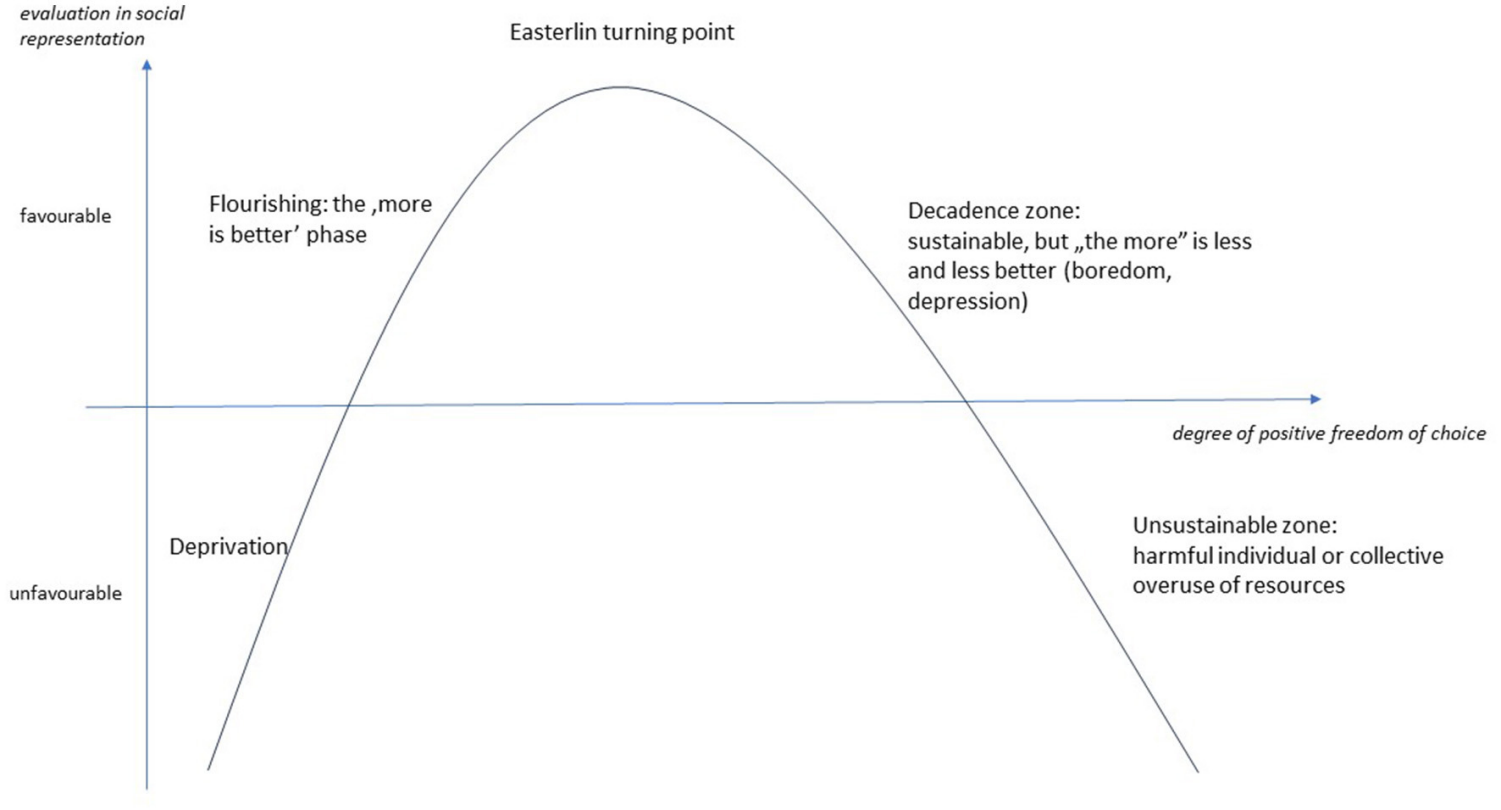
Relationship between evaluation in the social representation of wellbeing and the degree of positive freedom of choice.

The study of factors that influence wellbeing and influence positive freedom of choice is a high priority in academic research (30, 41, 52). However, we argue that the importance of the *degree* of positive freedom is less recognized due to “the more is better” bias presented earlier. However, a few arbitrary examples from wellbeing research illustrate that the degree of positive freedom of choice is not a negligible factor in wellbeing. This relationship is illustrated in Figure 1.

In our model, the absence or low level of positive freedom of choice is deprivation, which is clearly disadvantageous and negative. At this stage, all resources are still devoted to mere biological or societal survival, but even for that is not fully sufficient. In this phase, the lives of individuals and communities are on a limited trajectory, with a minimum of room for maneuver due to the necessities of survival. From a public health perspective, this zone receives particular attention, for example in the Social Determinants of Health (SDoH) model (34, 53).

The increase in positive freedom of choice is accompanied by an increasing amount of “surplus” energy, time, and resources in the lives of individuals and communities, which are not used for mere biological or societal survival, but also provide opportunities—in a relatively autonomous way—for the realization of other kinds of goals and aspirations. At this stage, the principle of 'the more is better' applies, with all its positive individual (flourishing) or collective consequences. In health sciences, the specificities of this zone can be found, for example, in the work of Antonovsky, especially in the context of general resistance resources (38).

However, based on the Easterlin paradox presented earlier (42), it is reasonable to assume that an increase in positive freedom of choice is only beneficial up to a certain level, and after a turning point, it may be neutral or even unfavorable.

If the positive freedom of choice exceeds the turning point, the individual or the community cannot or does not want to deal with the “excess” energy. In the absence of prospective goals, the increasing positive freedom of choice, and the growing general resistance resources, lead to increased boredom. The importance of the societal and economic problems resulting from boredom, and the importance of literate consumption in preventing these problems, was already shown by Scitovszky in the 1970s (54).

Ultimately, as the negative effects of over-indulgence in positive freedom become more pervasive, “the more is better” principle finally fails, and over-indulgence becomes a source of individual or social-societal crisis. According to Caplan (55), a crisis is a turning point after which life can no longer go on as before. It can lead to destruction or renewal, but a lifestyle that reflects a certain degree of positive freedom of choice causes increasing individual or collective harm and is not sustainable in the long term.

### 3.5 The negative freedom of choice factor of wellbeing

One of the cornerstones of our concept of wellbeing is that analyzing wellbeing only in terms of positive freedom of choice leads to significant distortions. Most of the current narratives on wellbeing present a one-sided picture, with an emphasis on growth driven by “the more is better” principle, and on the internal and external obstacles that can hinder growth despite our best intentions. Little attention is paid to the deliberate and conscious brakes and counterbalances that enable the growth of individual and collective wellbeing to be a controllable, manageable, and sustainable process, at both individual and collective levels. These negative preferences acting as a brake and counterbalance are necessary for the development of wellbeing to be a manageable process. To use an analogy, wellbeing is currently treated as a vehicle that can only accelerate and can only be stopped by traffic obstacles. Such a vehicle without brakes is useless in practice because it is unsuitable for safe driving.

**Figure 2:**
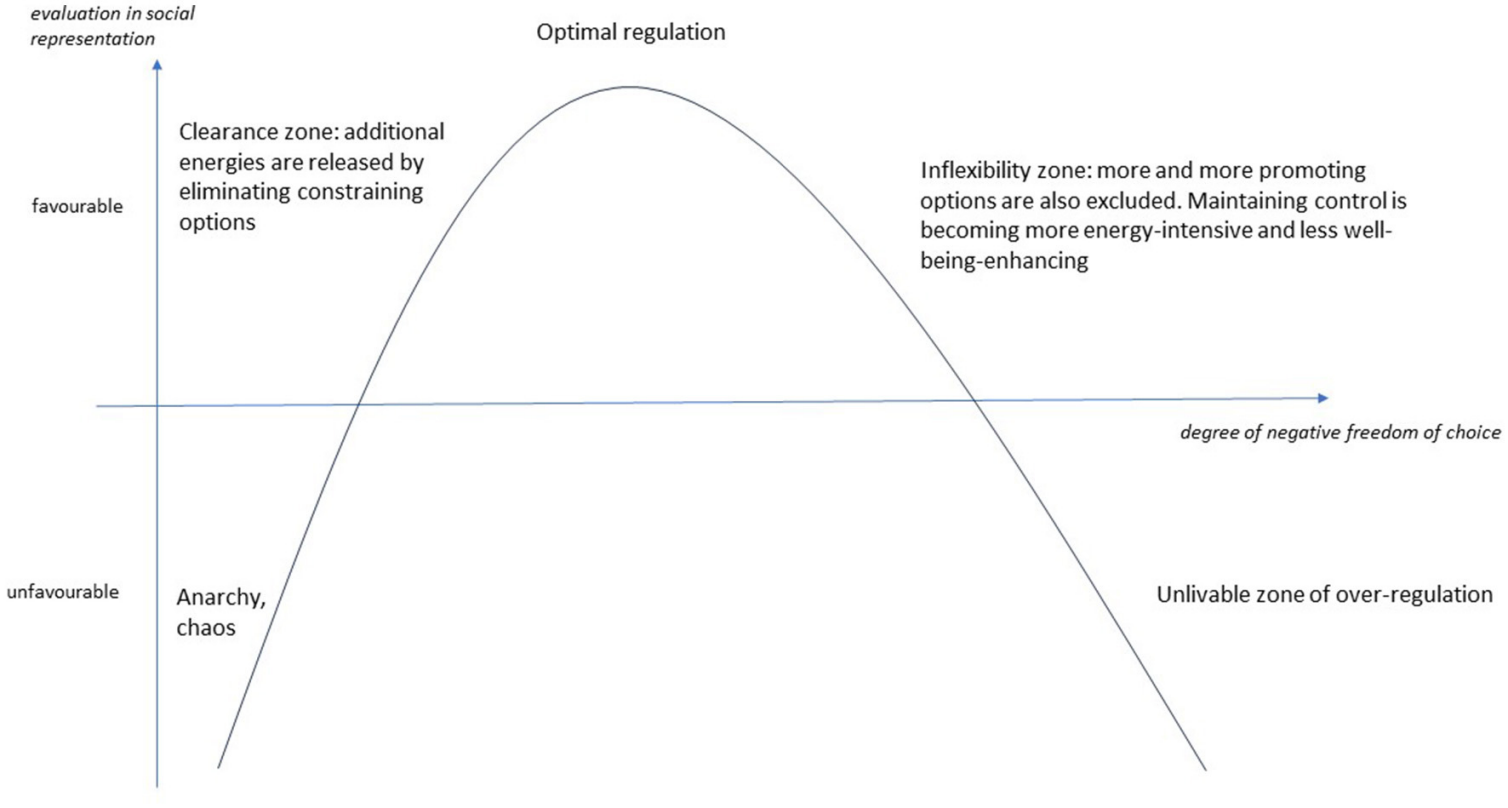
Relationship between evaluation in the social representation of wellbeing and the degree of negative freedom of choice.

Consequently, we also analyzed the association of the negative degrees of freedom of choice with wellbeing, and the results are summarized in Figure 2.

For understandable reasons, scientific narratives of wellbeing focus on the unlivable zone of over-regulation from societal aspect (53), because the entrenched principles of disenfranchisement, exploitation, and unequal opportunity are overtly threatening and dangerous to wellbeing. This phase can therefore be interpreted as a proliferation of negative preference structures.

But just as dangerous and threatening can be the absence of negative preferences, which we have marked on our diagram as the zone of anarchy and chaos, where “nothing is forbidden” and anyone can do anything and to anyone, anything can be done. The lack of stability and predictability derived from norms and rules is as threatening to wellbeing as the unlivable tyrannical over-regulation (39).

Negative freedom of choice can therefore have a positive aspect in terms of quality of life. Self-control, the “art” of saying no, etc., is about being able to commit to an individual or community negative preference. Even if our commitment means saying no to some of our positive preferences. Therefore, perseverance, determination, moderation, concentration are signs of experiencing a favorable range of negative freedom (41).

The importance of negative freedom of choice lies not only in the exclusion of decision options but also in the ability to delay immediate decisions and to consider long-term aspects (45). In our model, the clearance zone marks the point at which individuals and communities experience that “less can be better.” The individual and collective benefits of principles, norms, and rules are also reflected in the reduction of cognitive dissonance, less energy, and time needed to make decisions, by excluding certain options. This clarity increases wellbeing (46). In the optimal range of negative freedom of choice, we can exclude all relevant hindering factors and thus focus on the relevant promoting factors (56).

A life with too few options makes it impossible to set and achieve achievable goals. As the power to determine possibilities based on dogmas expands the scope of maneuvering, the chances of achieving goals increase, while dogmas limit the ability to adapt to the challenges of reality and thus succeed. In our concept, the inflexibility zone is reached when options that promote wellbeing are increasingly excluded. This is when rules and norms start to lose their original significance, and control for its own sake becomes more and more important. The intensification of negative freedom of choice becomes over-regulated when the prevalence of self-serving control makes life unlivable at an individual and/or collective level (57).

### 3.6 Wellbeing is characterized by a combination of positive and negative freedom of choice

**Figure 3:**
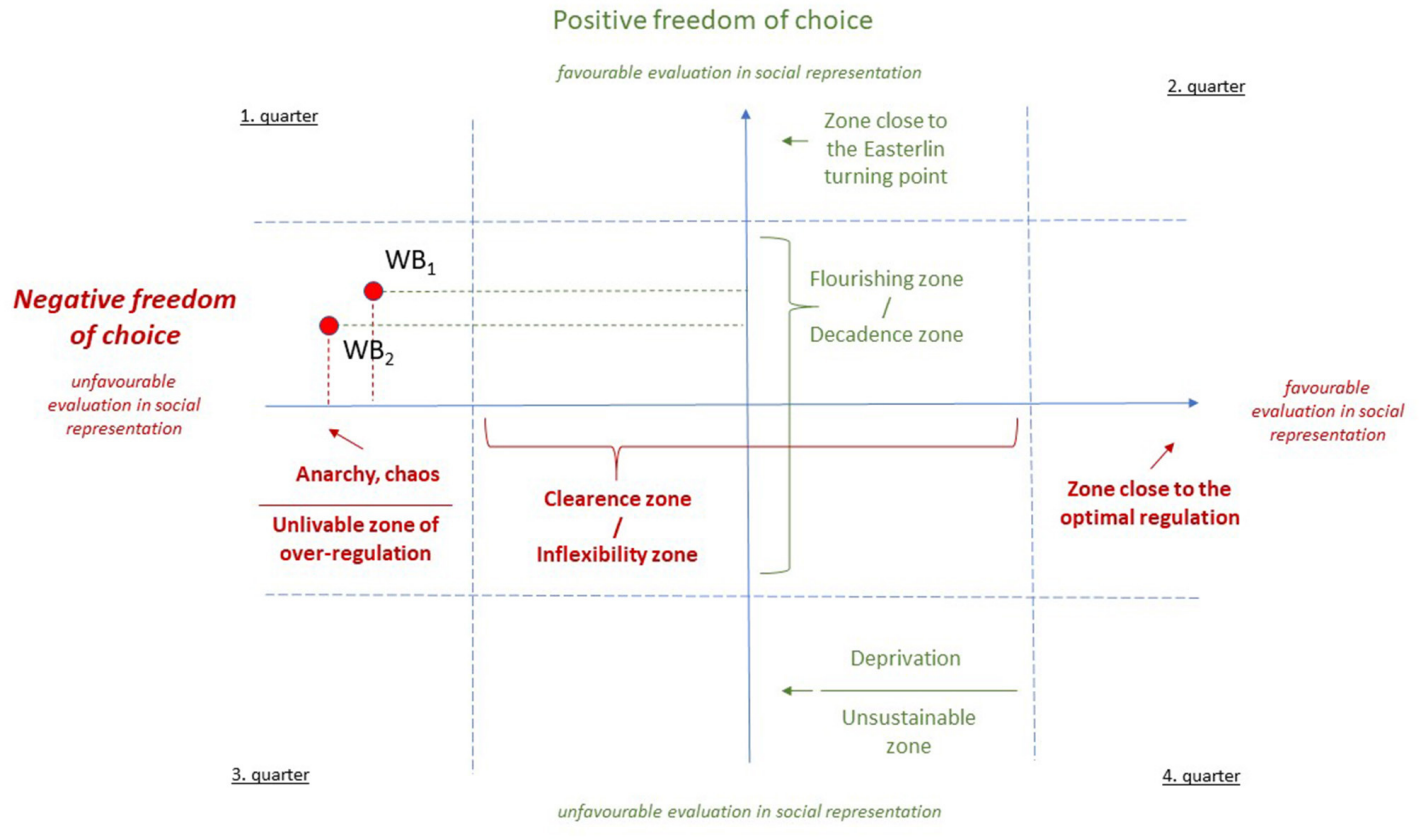
Coordinate system characterizing the social representation of wellbeing by the degree of positive and negative freedom of choice.

By placing the two types of *freedom of choice* on a single coordinate system based on their social representational valuation, the combination of positive and negative freedom of choice valuations can be analyzed. To represent the two-factor values together, the factors were treated as two intersecting surfaces and projected onto a two-dimensional coordinate system (see Figure 3).

In our concept, the two types of freedom of choice can be seen as independent of each other. For example, the flourishing zone of positive freedom of choice can be combined with the anarchy and chaos of negative freedom of choice [e.g., internet use by young people in the USA (58)], and also with unlivable over-regulation [e.g., internet use in China (27)]. To illustrate the explanatory power of our meta-theory, we have also plotted it with the two hypothetical points in Figure 3: WB1, as the US situation, and WB2, as the Chinese situation.

Our concept can be applied at all levels of analysis relevant to wellbeing: individual, family, organizational, local community, regional, societal, and humankind. Furthermore, in addition to the concept of wellbeing, it can also be used as a framework for analyzing the social representation of a specific topic (e.g., internet use, healthy nutrition, etc.) or subject area (e.g., quality of life). Research on the social representation and positioning of wellbeing will require a combination of qualitative and quantitative research methods. However, we would like to emphasize that the practical relevance of our concept is primarily that it allows us to track the dynamic changes in the combination of dimensions used in the social representation of wellbeing and the positional evolution of wellbeing at the given level of analysis, whether for a specific individual, family, workplace, school, region, or society.

### 3.7 Health in the wellbeing meta-theory

In our wellbeing meta-theory, *health* is a specific aspect of the social representation of wellbeing and the wellbeing positioning based on it, which includes aspects of physical, mental (cognitive and emotional), social (social and societal), and spiritual functionality and functioning of *individuals* and their evaluation. This aspect can shape both positive and negative freedom of choice factors of wellbeing.

*Health is therefore an important part of the meta-theory of wellbeing, but not the whole. The question of what we use our functionality for is outside the scope of health*. It is difficult to understand from the perspective of a health science narrative, but much more meaningful from other narratives of wellbeing, that there are individual and collective choices where health is *not* the priority. At an individual level, people in dangerous and self-sacrificing professions, such as police officers, soldiers, and health professionals, have chosen to prioritize their vocation and profession over preserving their health. But even in the most mundane tasks and situations, we often focus more on our goals and consciously do not prioritize maintaining our functionality.

These individual choices are also reflected at a societal level. The Lalonde report (22) already pointed out that in a significant number of cases, the healthcare system is now facing the harmful and significant consequences of not giving sufficient priority to maintaining health for one reason or another. One of the main reasons for this is that societies prioritize the achievement of their current political, economic, and cultural goals, and the preservation of the functionality of the population is relegated to the bottom of the priority list. Both the health and social care systems, as represented in the wellbeing meta-theory, tend to concentrate their efforts on the areas that are rated as unfavorable in terms of both positive and negative freedom of choice—the third quarter—and focus on mitigating the damage here (cf. Figure 3).

The health and social care system performs necessary and important societal functions. However, as shown earlier, this does not affect the positive and negative aspects of freedom of choice that are beneficial to the quality of life and does not trigger the need for deliberate and direct interventions to improve wellbeing and health. The health promotion efforts outlined in the introduction (23) or Antonovsky's salutogenetic health model (38) already reflect the recognition that real progress in terms of wellbeing and health in society goes beyond damage control, i.e., takes place in the second quarter of the wellbeing coordinate system (cf. Figure 3). This is essentially the public health paradox we outlined at the beginning of our study. The societal challenges to be addressed by public health are considered in the 3rd quarter, while the tools and methods needed for greater efficiency can be derived from the 2nd quarter, which focuses directly on improving wellbeing. However, this is beyond the scope of the public health system, as promoting the individual, family, local community, school, workplace, and policy changes needed to achieve the goals and priorities is outside the scope of public health.

*The advantage of our wellbeing meta-theory may be that it provides a framework for understanding and developing social representations of the combination of positive and negative freedom of choice in the quality of everyday life and the wellbeing positioning based on them, in which all the different disciplines, professions and social subsystems can play a role so that none of them is in a privileged position*.

In our study, we argue that the development and operation of a new institutional system—a so-called Public Wellbeing System (PWS)—*alongside* the current health and social care system, dedicated to the promotion of wellbeing and characterized by the above meta-theory of wellbeing, could provide an opportunity to overcome the public health paradox.

## 4 The promotion of wellbeing: the public wellbeing system

Public Wellbeing System refers to a system of governmental and non-governmental organizations at local, regional and national level whose primary strategic mission is to improve wellbeing and health, mainly by identifying and representing the interests of different social strata and groups using scientific methods and by identifying and coordinating the opportunities for wellbeing development in different sectors. The arguments for developing and operating a public wellbeing system (PWS) are summarized below.

*1. Exploiting the public health potential of the wellbeing brand more effectively*. We believe that one of the obstacles to the further development of public health is, paradoxically, the word 'health' itself. As the word is still mostly used in the sense of ill health, the majority of professionals and the general public understand the term mainly in a biomedical connotation. *Non-medical professionals* may well have the question, why and how should they be involved if they are not doctors or health professionals? Why and how should they be involved if their work is not about preventing and curing disease? This can be a barrier not only to communication but also to interprofessional cooperation. Wellbeing as a new social marketing brand can provide opportunities to better communicate a broader view of health while creating new opportunities for inter-professional cooperation.

*2. PWS also has the potential to bring about the qualitative change in health that is needed to move beyond the inherent limitations of the Health in All Policies Directive*. Since the Ottawa Charter, WHO has advocated the principle that health should be integrated into all aspects of life. Grossman and Scala (59) aptly put it that in society there is a social subsystem for illness (the health-care system), but no such subsystem for health. Health must therefore be integrated into all social subsystems. The development of a new social subsystem can bring about a qualitative change in this area, which promises more results than the continuation of the current public health and health promotion strategies, extended with “wellbeing” (29), but unchanged in approach.

*3. PWS can be a credible representative of health promotion in society through its organizational goals and organizational culture focused on wellbeing and health promotion*.

The dichotomous concept of health, based on discrete categories of illness and health, is not only a lay concept but is also reflected in current social subsystems. “Present health-care systems focus on illness. The treatment of illness is not only better organized but also apparently easier to organize than health. […] Organizations are made to solve problems; illness is a problem, but health is not” [(59), p. 26]. The Ottawa Charter (23) was born out of the need for a paradigm shift in healthcare. While risk management is part of a goal-driven, salutogenetic (38) approach to health promotion, it does not replace the need to develop a 'target system' for real development. A systems approach to health promotion encompasses medical thinking and the biomedical framework for disease prevention and treatment but also goes beyond this to where people “learn, work, play and love” in everyday life (23). For the health-care system, health promotion is a secondary task compared to curing, and therefore it cannot be expected to fully represent its approach and values in society.

It is worth noting that the need for a renewed focus on wellbeing rather than health, including the fundamentals, was raised by Prilleltensky as early as 2005: “It is high time for a paradigm shift in health and human services, … only a new approach that focuses on strengths, prevention, empowerment, and community conditions can make considerable progress toward the achievement of wellbeing for all.” [(40), p. 53]. At present, none of the existing social sub-systems can take on this task without the risk of distorting this challenge in the direction of their existing social tasks. For this reason have we in our introduction included what we hope will be constructive criticism of the current WHO core documents on wellbeing (23, 29). And this justifies the creation of a social subsystem whose main social task is to promote wellbeing and health.

*4. We already have some insight into the main guidelines and methods of PWS*. The main task of a public wellbeing system can be the preparation, implementation, and evaluation of coordinated multisectoral activities to raise the level of wellbeing. This will enable a more targeted and efficient use of societal resources. Public wellbeing is an issue that can be used to mobilize all sectors of society for wellbeing and can be aligned with a wide range of political and social interests. The core activity of PWS is therefore the systemic facilitation of individual, community, and societal developments that promote wellbeing (and health promotion within this). This also requires an intervention methodology that is primarily based on the involvement and participation of those concerned, i.e., it focuses on cooperation and co-creation rather than care.

*5. PWS is currently a utopia, but it is a utopia toward which it is worth taking steps in the present, in which public health can play a key role*. In the case of PWS, it is also worth considering Gall's “law,” which states that functional complex systems always evolve from a simple but functional version, through evolutionary development (60). As a consequence, there is little chance that a complex system such as PWS can be developed in its entirety from “behind a desk.” But promising steps can already be taken, and are essential if a new social subsystem is to emerge.

## 5 Discussion

We have argued in our study that the main reason for the paradoxical situation of public health is that the societal challenges for which it is responsible can only be partially addressed by a public health system based on the perspective, priorities, and scope of the health-care system.

The strength of our wellbeing meta-theory is that it can be used to identify “game-changer” interventions that can lead to tangible changes in the social representation of wellbeing, can be financed at the given level of socio-economic development and have a significant public health relevance. Just one example. The issue of food aid for the hungry in a society is undoubtedly an important social policy issue with high public health relevance. But what can explain the fact that even at a theoretical level we are not addressing the question of how to make the possibility of a healthy nutrition available to all as a basic right? Public health, building on its health promotion tradition, could very well be a facilitator of such projects pointing in this direction. The application of our meta-theory, and ultimately the operation of PWS, can highlight such opportunities and can be an effective tool for preparing governmental and non-governmental decisions, for assessing state and needs at municipal and organizational levels, and for facilitating intersectoral cooperation.

A limitation of our study is the complexity of the wellbeing meta-theory and PWS, and the lack of sophisticated interdisciplinary methodology. With the meta-theory of wellbeing we have developed in this paper, we have attempted to outline the possibility of a model that provides a common denominator for the diverse narratives of wellbeing in different disciplines. In developing our meta-theory, we have made a conscious effort to draw on as many disciplinary perspectives as possible, but not to give any one narrative more weight than the others. The *diversity in the content* of social representations of the combination of positive and negative freedom of choice in the quality of everyday life cannot be captured from the perspective of a single discipline. This task requires the synthesis of results from many disciplines, which is not possible without a common denominator, an overarching meta-theory.

An important element of our perspective is that the health science narrative of wellbeing is only one of many valuable narratives of wellbeing. We therefore see a need for public health to do more than it has so far to develop an equal partnership with other disciplines in the field of wellbeing.

A striking sign of this endeavor, and at the same time an indispensable one for the development of wellbeing societies, would be the development of a systemically mediated, development-oriented institutional system based on the principles of participation, which is dedicated to the development of wellbeing and is a coherent network of organizations with such goals and culture. In particular, the experience of health promotion in line with the spirit of the Ottawa Charter can be very useful.

According to our meta-theory, the development of wellbeing cannot effectively be subordinated to the promotion of health, because health is an important part of human wellbeing, but not the whole of it. But promoting wellbeing is also an important public health issue because it offers an opportunity to organize our health knowledge more effectively in society, without the risk of narrowing the focus back to health and the prevention and treatment of disease.
